# Salinity and Mulching Effects on Nutrition and Production of Grafted Sour Passion Fruit

**DOI:** 10.3390/plants12051035

**Published:** 2023-02-24

**Authors:** Antônio Gustavo de Luna Souto, Lourival Ferreira Cavalcante, Edinete Nunes de Melo, Ítalo Herbert Lucena Cavalcante, Roberto Ítalo Lima da Silva, Geovani Soares de Lima, Hans Raj Gheyi, Walter Esfrain Pereira, Vespasiano Borges de Paiva Neto, Carlos Jardel Andrade de Oliveira, Francisco de Oliveira Mesquita

**Affiliations:** 1 Postgraduate Program in Plant Production, Federal University of the São Francisco Valley, Petrolina 56300-000, Brazil; 2 Postgraduate Program in Agronomy, Federal University of Paraiba, Areia 58397-000, Brazil; 3 Department of Plant Science, Federal University of Paraíba, Areia 58397-000, Brazil; 4 Postgraduate Program in Agricultural Engineering, Federal University of Campina Grande, Campina Grande 58429-900, Brazil; 5 Department of Soils and Mineralogy, National Institute of the Semiarid, Campina Grande 58434-700, Brazil

**Keywords:** *Passiflora edulis* f. flavicarpa Degener, abiotic stress, rootstock, plastic film, mineral composition, yield fruit

## Abstract

The Brazilian semiarid region stands out in terms of sour passion fruit production. Local climatic conditions (high air temperature and low rainfall), combined with its soil properties (rich in soluble salts), increase salinity effects on plants. This study was carried out in the experimental area “Macaquinhos” in Remígio-Paraíba (Brazil). The aim of this research was to evaluate the effect of mulching on grafted sour passion fruit under irrigation with moderately saline water. The experiment was conducted in split-plots in a 2 × (2 × 2) factorial scheme to evaluate the effects of the combination of irrigation water salinity of 0.5 dS m^−1^ (control) and 4.5 dS m^−1^ (main plot), passion fruit propagated by seed and grafted onto *Passiflora cincinnata*, with and without mulching (subplots), with four replicates and three plants per plot. The foliar Na concentration in grafted plants was 90.9% less than that of plants propagated via seeds; however, it did not affect fruit production. Plastic mulching, by reducing the absorption of toxic salts and promoting greater absorption of nutrients, contributed to greater production of sour passion fruit. Under irrigation with moderately saline water, the plastic film in the soil and seed propagation promote higher production of sour passion fruit.

## 1. Introduction

In arid and semi-arid regions, soil salinity and irrigation management have a direct relationship and affect plants as a function of soluble salt concentrations and compositions of water sources [1]. Soil salinity is affected by irrigation with saline water from dams or artesian wells and saline wastewater (brine) discharged by desalination plants and process industries such as oil and gas, textile, leather, food, dairy, agriculture, and pharmaceutical industries [2,3,4]. Under high salt concentrations, crop yields may be severely affected by water deficit due to low soil-solution osmotic potential (osmotic effect) and by nutritional imbalance, which may be induced by salinity associated with excessive absorption of toxic ions (Na^+^ and Cl^−^) or nutrient availability, transport, or partition within the plant [5,6,7].

In Brazil, high saline levels in the soil or irrigation water are more common in semi-arid regions of the northeast regions due to low rainfall and high air temperatures [8,9]. This region produces about 71.2% of the Brazilian sour passion fruit (*Passiflora edulis* f. flavicarpa Degener) [10]. The water sources available often have moderate to high concentrations of soluble salts, which contribute to soil degradation, nutritional imbalance, and yields below 10.0 t ha^−1^ [10,11].

According to the threshold salinity of the crops, most varieties of sour passion fruit cultivated behave as salt-sensitive species, with significant reductions in their yields from irrigation water salinity, leading to electrical conductivity of irrigation water (ECiw) = 1.3 dS m^−1^ [11,12]. In addition, the greater or lesser sensitivity of plants to salt stress varies depending on differences in climate, soil, and cultural management factors in each growing region [13]. Recent studies have shown that wild species of *Passiflora* ssp. have greater tolerance to salinity than the sour passion fruit [14,15]. Therefore, they can be used as the rootstock of commercial species for cultivation in saline areas [9,16]. The need for information on the tolerance and mineral nutrition of plants in saline zones, and therefore, on the impact of salinity on fruit production, has a direct economic impact [17].

The excess of toxic elements in cells, such as sodium (Na^+^) and chloride (Cl^−^) ions, increases oxidative stress by increasing the production of reactive oxygen species (ROS), which causes damage to proteins, lipids, and nucleic acids [18]. Over time, some species have developed tolerance mechanisms to acclimate to saline environments, such as exclusion, compartmentalization of toxic ions, and preference for absorption of essential elements by plants, called ionic homeostasis [19]. Salt-tolerant rootstocks reduce leaf concentrations of Na^+^ and Cl^−^ in melons (*Cucumis melo*) [20] and citrus fruits—*Citrus macrophylla* and *Citrus reticulata* [21], reducing their absorption by roots [7,22]. Such tolerant species also maintain essential elements, such as potassium, calcium, and magnesium, at adequate levels in leaves [23,24]. These results are crucial, since the decreasing order of nutritional demand of sour passion fruit is N > K > Ca > S > P > Mg, as reported by [25].

Another alternative to mitigate salinity effects on plants is plastic mulching (PM) on the soil surface. The technique is often used in agriculture of semi-arid regions to promote an adequate soil microclimate [26,27], favoring water-and nutrient-use efficiencies [28,29,30]. PM benefits are undeniable for arid and semi-arid areas affected by salinity problems, where the evaporative demand is high and soil and water naturally have high levels of soluble salts [31,32] which migrate by the capillary rise from deeper layers to the surface. Therefore, some studies have shown that PM reduces salinity within the root-zone, increasing fruit yields of species irrigated with saline water, as observed by [31] for grapevines (*Vitis* sp.) and by [33] for raspberries (*Rubus idaues*).

Grafting has been used to induce abiotic stress tolerance in several fruit species [21,22,23,24]. However, such a propagation method has progressed little for sour passion fruit, despite the salt-tolerant wild species [9,10,11]. Plastic mulching has recently been used in the exploitation of fruit species. According to [30], studies still lack progress in different edaphoclimatic conditions. There are gaps to be filled regarding the production benefits in several fruit species that are mainly irrigated with saline water. This study hypothesizes that the use of plastic mulching and the grafting technique with wild species of Passiflora, respectively, reduce the accumulation of salts in the root zone of the soil and increase the selectivity of absorption of essential elements to toxic ions (Na^+^ and Cl^−^), influencing the nutrition and productivity of sour passion fruit. Therefore, this study aimed to evaluate saline water and plastic mulching effects on the nutritional status and fruit production of sour passion fruit grafted on *Passiflora cincinnata*.

## 2. Results

### 2.1. Macronutrients

Regarding leaf concentrations of macronutrients, sour passion fruit plants responded differently to sources of variation (Table 1). While Ca responded to the interaction water salinity (WS) × propagation (Pg) × plastic mulching (PM), P levels were affected by the interaction of Pg × PM. Leaf K concentrations responded to interactions of WS × Pg, WS × PM, and Pg × PM. Leaf concentrations of Mg were influenced by the interactions of WS × Pg and Pg × PM, while leaf S concentrations were influenced by the interaction of WS × PM. Finally, N responded to PM application.

**Figure 1:**
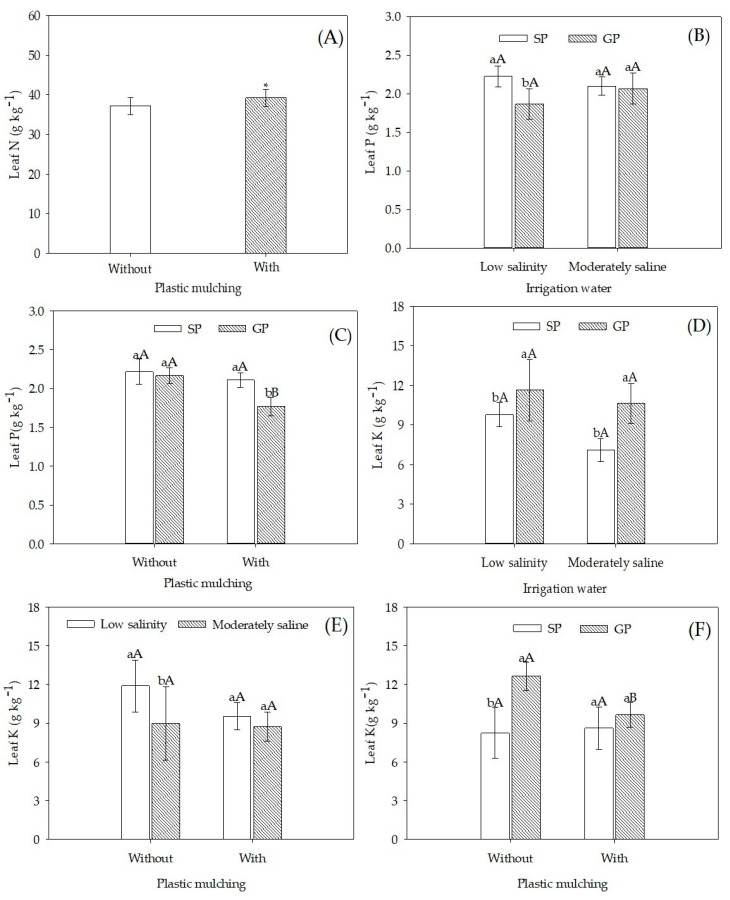
Concentration of macronutrients in leaves of sour passion fruit by seed-propagated and grafted propagated irrigated with low salinity and moderately saline waters with and without plastic mulching. (**A**) N concentration of sour passion fruit in mulched soil; (**B**) P concentration of seed-propagated (SP) and grafted propagated (GP) on *P. cincinnata* irrigated with low and moderately saline water; (**C**) K concentration of seed-propagated (SP) and grafted propagated (GP) on *P. cincinnata* in soil without and with plastic mulching; (**D**) K concentration of seed-propagated (SP) and grafted propagated (GP) on *P. cincinnata* irrigated with low salinity and moderately saline water; (**E**) K concentration in leaves of sour passion fruit irrigated with low salinity and moderately saline water and in soil without and with mulching plastic; (**F**) K concentration of seed-propagated (SP) and grafted propagated (GP) on *P. cincinnata* fruit in soil without and with mulching plastic. Vertical bars represent the standard error of the mean (*n* = 4). Bars with an asterisk (*) differ from each other for soil with and without plastic mulching by the F-test (*p* > 0.05) (**A**). Bars with the same lower-case letter are similar for soil with and without plastic mulching (**C**,**E**,**F**) or for low salinity and moderately saline irrigation water (**B**,**D**) by the F-test (*p* > 0.05). Bars with the same uppercase letter are similar for seed propagation and grafting (**B**–**D**,**F**) or low salinity and moderately saline irrigation water (**E**) by the F-test (*p* > 0.05).

Soil plastic mulching enhanced N concentrations in leaves from 37.3 to 39.3 g kg^−1^ (Figure 1A), representing an increase of 5.36%. Figure 1B indicates no difference in P leaf concentrations between sour passion fruits irrigated with low salinity and moderately saline water. However, in plants irrigated with low salinity water, SP plants showed a P concentration in leaves that was 19.4% higher than the GP seedlings. A higher P concentration was also verified in grafted plants grown in mulched soil (Figure 1C), with a P concentration 19.3% higher than non-grafted plants.

Irrigation water salinity did not affect the leaf concentration of K in either SP or GP plants (Figure 1D). However, when plants were irrigated with moderately saline water, K leaf concentrations were 50.4% higher in GP than in SP plants. Figure 1F shows that leaf K concentration in GP plants grown in non-mulched soil was higher than in mulched soil. Under non-mulched treatment, the lack of protection against water loss promoted a leaf K concentration that was 57% higher in grafted plants than in SP. Leaf concentrations of K did not differ between passion fruits grown in mulched and non-mulched soil (Figure 1E). However, an irrigation water salinity of 4.5 dS m^−1^ reduced leaf K concentration by 24.4% in plants grown in non-mulched soil but did not affect plants in mulched soil.

**Figure 2:**
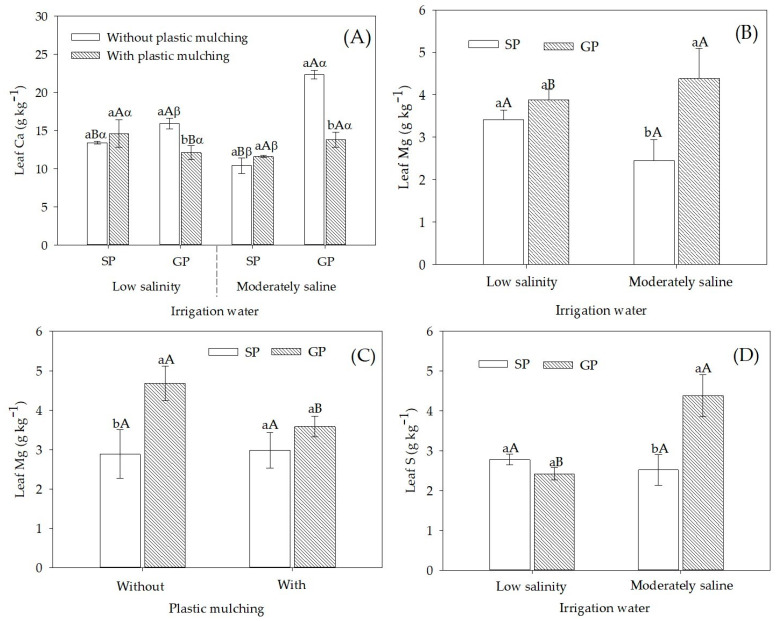
Concentration of macronutrients in leaves of sour passion fruit by seed-propagated and grafted propagated irrigated with low salinity and moderately saline waters with and without plastic mulching. (**A**) Ca concentration in sour passion fruit seed-propagated (SP) and grafted-propagated (GP) on *Passiflora cincinnata* irrigated with low salinity and moderately saline water and in soil without and with mulching plastic; (**B**) Mg concentration in sour passion fruit seed-propagated (SP) and grafted-propagated (GP) on *Passiflora cincinnata* irrigated with low salinity and moderately saline water; (**C**) Mg concentration in sour passion fruit seed-propagated (SP) and grafted-propagated (GP) on *Passiflora cincinnata* in soil without and with plastic mulching, and (**D**) S concentration in sour passion fruit seed-propagated (SP) and grafted-propagated (GP) on *Passiflora cincinnata* irrigated with low salinity and moderately saline water. Vertical bars represent the standard error of the mean (n = 4). Bars with the same lower-case letter are similar for soil without and with plastic mulching (**A**) or for seed propagation and grafting (**B**–**D**) by the F-test (*p* > 0.05). Bars with the same uppercase letter are similar for seed propagation and grafting (**A**) or low salinity and moderately saline irrigation water (**B**,**D**) or soil without and with plastic mulching (**D**) by the F-test (*p* > 0.05). Bars with the same Greek letter are similar for low salinity and moderately saline irrigation water (**A**) by the F-test (*p* > 0.05).

For SP plants, Ca concentrations in the leaf did not differ between plants grown in mulched and non-mulched soil, regardless of the irrigation water salinity level (Figure 2A). However, for GP plants, the highest leaf Ca concentrations were observed in plants grown in non-mulched soil, especially for plants irrigated with moderately saline water. These GP plants showed leaf Ca concentrations that were 66.4% higher than SP plants irrigated with low salinity water and 114.4% higher than SP irrigated with moderately saline water.

Moderately saline water irrigation in GP plants increased the leaf Mg concentration (Figure 2B). Sour passion fruit plants grafted on *P. cincinnata* increased leaf Mg concentration by 78.8% when compared to SP plants irrigated with 4.5 dS m^−1^ water. On the other hand, plastic mulching caused no significant effect on leaf Mg concentration in SP. However, GP plants grown in non-mulched soil had a higher nutrient concentration than those that were grown in in mulched soil (Figure 2C). When comparing propagation forms under both soil mulching conditions, GP had a leaf Mg concentration that was 62.5% higher than SP. When irrigated with moderately saline water, the sour passion fruit grafted propagated showed a higher S concentration than the plants seed-propagated (Figure 2D). Furthermore, under irrigation with moderately saline water, the sour passion fruit showed an increase in S concentration of 80.1% compared to irrigation with low salinity water.

### 2.2. Micronutrients and Sodium

Leaf concentrations of Cu, Fe, Mn, Zn, and Na were influenced by the interaction of WS × Pg × PM (Table 2). In addition, the interaction of Pg × PM affected leaf B concentrations, while Cl responded to the interaction of WS × PM.

**Figure 3:**
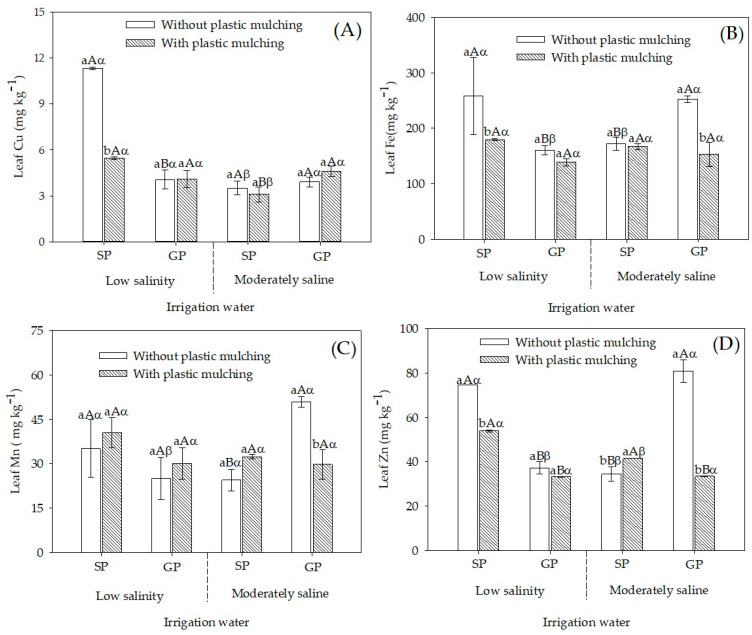
Concentration of micronutrients in leaves of sour passion fruit by seed-propagated and grafted propagated irrigated with low salinity and moderately saline waters with and without plastic mulching. (**A**) Cu, (**B**) Fe, (**C**) Mn, (**D**) Zn concentration in sour passion fruit seed-propagated (SP) and grafted propagated (GP) on *Passiflora cincinnata* irrigated with low salinity and moderately saline water in soil with and without plastic mulching. Vertical bars represent the standard error of the mean (n = 4). Bars with the same lower-case letter are similar for soil without and with plastic mulching by the F-test (*p* > 0.05). Bars with the same uppercase letter are similar for seed propagation and grafting by the F-test (*p* > 0.05). Bars with the same Greek letter are similar for irrigation with low salinity and moderately saline water by the F-test (*p* > 0.05).

The highest leaf Cu concentration was observed in SP sour passion fruit irrigated with low salinity water and grown without mulching, with an increase of 178.32% compared to GP plants (Figure 3A). However, under irrigation with 4.5 dS m^−1^ water and in mulched soil, the leaf Cu concentration was 48.4% higher in GP plants than in SP plants.

Under irrigation with low salinity water, leaf Fe and Zn concentrations were higher in seed-propagated plants in non-mulched soil; concentrations were 61% and 100.2% superior to those of grafted-propagated plants, respectively (Figure 3B,D). However, no significant (*p* > 0.05) difference was observed for Mn concentration (Figure 3C). The opposite behavior was observed in plants under moderately saline water irrigation, but with grafted plants: the leaf Fe, Mn, and Zn concentrations were higher than those of seed-propagated plants grown in soil without mulching, with increases of 46.6, 108.1, and 134.8%, respectively.

**Figure 4:**
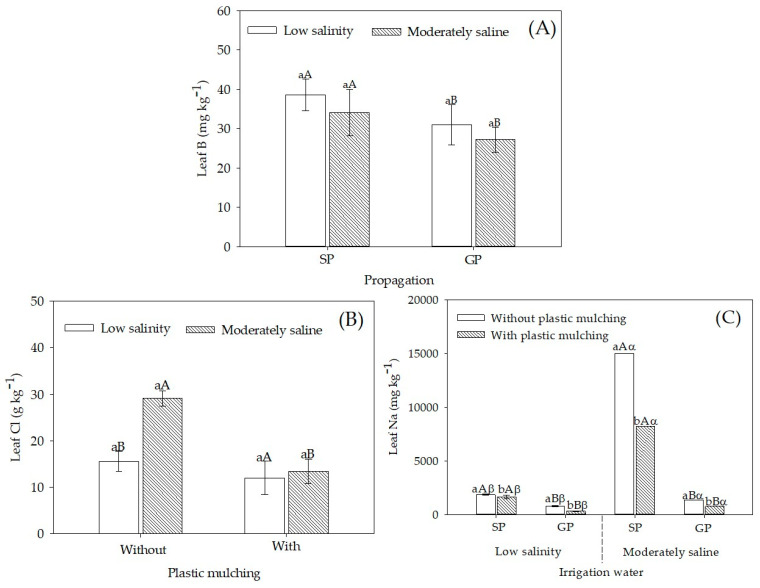
Concentration of micronutrients (boron and chlorine) and sodium in leaves of sour passion fruit by seed-propagated and grafted propagated, irrigated with low salinity and moderately saline waters, with and without plastic mulching. (**A**) B concentration in sour passion fruit seed-propagated (SP) and grafted propagated (GP) on *Passiflora cincinnata* irrigated with low salinity and moderately saline water; (**B**) Cl concentration in sour passion fruit irrigated with low salinity and moderately saline water in soil with and without plastic mulching; (**C**) Na concentration in sour passion fruit seed-propagated (SP) and grafted propagated (GP) on *Passiflora cincinnata* irrigated with low salinity and moderately saline water in soil with and without plastic mulching. Vertical bars represent the standard error of the mean (n = 4). Bars with the same lower-case letter are similar for soil with and without plastic mulching (**A**) or low salinity and moderately saline irrigation water (**B**,**C**) by the F-test (*p* > 0.05). Bars with the same uppercase letter are similar for soil with and without plastic mulching (**B**) or seed propagation and grafting (**A**,**C**) by the F-test (*p* > 0.05). Bars with the same Greek letter are similar for irrigation with low saline and moderate saline water (**A**) by the F-test (*p* > 0.05).

Leaf B concentrations did not differ significantly between irrigation with low salinity and moderately saline water (Figure 4A). However, SP plants showed higher foliar B concentration than GP plants, with 24.4% and 25.7% increments in plants irrigated with 0.5 and 4.5 dS m^−1^ water, respectively.

Sour passion fruit irrigated with moderately saline water had higher leaf Cl concentration, but plastic mulching considerably reduced its concentrations in leaf tissues (Figure 4B). Mulching reduced leaf Na concentration in the sour passion fruit, regardless of the irrigation water salinity (Figure 4C). Moreover, GP plants under salt stress had leaf Na concentrations similar to those of plants irrigated with water at 0.5 dS m^−1^. Such findings are significant compared to SP plants under moderately saline water irrigation (14,982.2 mg kg^−1^), in which the Na concentration was 996.7% higher than that in GP (1366.1 mg kg^−1^).

### 2.3. Production of Fruits per Plant

**Figure 5:**
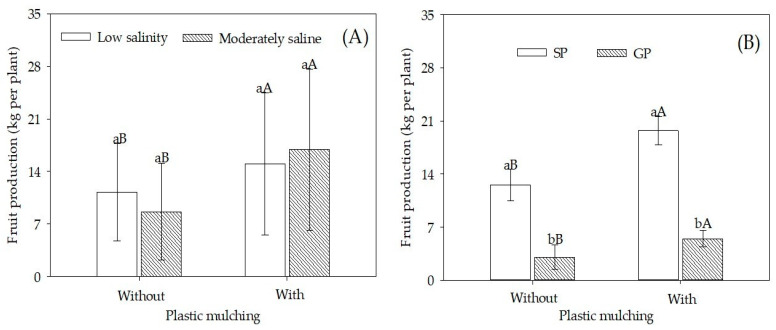
Fruit production of sour passion fruit seed-propagated and grafted propagated irrigated with low salinity and moderately saline waters and with and without plastic mulching. (**A**) Fruit production of sour passion fruit irrigated with low salinity and moderately saline water in soil with and without plastic mulching; (**B**) Fruit production of sour passion fruit seed-propagated (SP) and grafted propagated (GP) on *Passiflora cincinnata* in soil with and without plastic mulching. Vertical bars represent the standard error of the mean (n = 4). Bars with the same lower-case letter are similar for soil without and with plastic mulching (**A**,**B**) and bars with the same uppercase letter are similar for low salinity and moderately saline irrigation water (**A**) or seed propagation and grafting (**B**) by the F-test (*p* > 0.05).

The fruit yield per plant was affected by the interactions of WS × PM (F = 70.62; *p* = 0.0001) and Pg × PM (F = 37.96; *p* = 0.0001). The salinity of the irrigation water did not affect the production of sour passion fruit (Figure 5A). In addition, the use of plastic cover in the soil increased fruit production from 11.26 to 15.03 kg per plant (low salinity water) and from 8.65 to 16.93 kg per plant (moderately saline water). However, SP sour passion fruit showed higher production than the GP ones, mainly in plants grown in mulched soil, with an increase of 259.5% (Figure 5B). Soil protection with mulching increased production per plant by 57.1% in SP and 78.7% in GP plants.

## 3. Discussion

Irrigation with moderately saline water had no significant effect on the leaf N concentration in passion fruit; this agrees with the results presented by [34] for the same crop under irrigation with the same type of water. Increases in leaf N concentration in yellow passion fruit grown in soil under plastic mulching (Figure 1A) can be attributed to decreases in water losses by evaporation and N losses by leaching. These reductions are due to improvements in thermal amplitude and soil moisture, increasing N absorption and nutrient-use efficiency by plants [28,29,30]. Nevertheless, sour passion fruit plants had an adequate N concentration in both treatments, within the adequate range of 36.0 to 46.0 g kg^−1^ [35].

Figure 1B,C show that only grafted sour passion fruits under irrigation with low salinity water and in mulched soil had leaf P concentrations outside the recommended range of 2.0–3.0 g kg^−1^ [35]. Zucarelli et al. [36] verified the same trend in the purple passion fruit grafted on *Passiflora cincinnata*, which had leaf P concentrations lower than non-grafted plants. Moreover, fertigation with potassium sulfate can reduce P absorption due to ionic antagonism between H_2_PO_4_^−^ and SO_4_^2−^ ions [6].

The use of *Passiflora cincinnata* as rootstock for sour passion fruit increased tolerance or adaptability to salinity and efficiency in K acquisition (Figure 1D) regardless of the mulching condition (Figure 1F), maintaining sufficient leaf K concentrations. The higher K absorption capacity of plants grafted on *P. cincinnata* tends to restrict the absorption and transport of toxic ions (Na^+^ and Cl^−^) of the irrigation water, as reported by [24] in grafted and non-grafted pomegranate (*Punica granatum* L.) under irrigation with 7.0 dS m^−1^ water.

The benefits of mulching on the soil by reducing heat and increasing humidity enhanced K absorption and accumulation in sour passion fruit leaves (Figure 1E); this impacted soil microbiota, which in turn increased K availability in plants through decomposition and cycling of nutrients in the soil [29,37]. Despite the increases, sour passion fruit plants were deficient in K, since the sufficiency range is between 24.0 and 32.0 g kg^−1^ [35].

Leaf Ca, Mg, and S concentrations were higher in GP than in SP plants, mainly under moderately saline water irrigation (Figure 2). In several crops, tolerant species have been used as rootstocks for salt sensitive commercial species, such as tomatoes—*Solanum lycopersicum* [5], melon—*Cucumis melo* [20,23], pumpkins—*Cucurbita ficifolia, Cucurbita moschata* L. landraces [6,7], and pomegranate [24].

As rootstock, *P. cincinnata* provided salt tolerance in sour passion fruit by selective absorption of nutrients and reduction in absorption and transport of Na^+^ and Cl^−^ ions, in addition to accumulation and compartmentalization of toxic ions in root cells [7,8,9,10,11,12,13,14,15,16,17,18,19,20,21,22,23,24]. Under saline conditions, sour passion fruit grafted on *P. cincinnata* was properly supplied with Ca, Mg, and S, according to their crop sufficiency ranges of 17–28 g kg^−1^, 2.1 g kg^−1^, and 4.4 g kg^−1^, respectively [35].

In the present study, the employment of *P. cincinnata* as rootstock increased absorption and leaf concentrations of micronutrients (Cu, Fe, Mn, and Zn) in sour passion fruit under salt stress (Figure 3). In cucumbers irrigated with 5.7 dS m^−1^ water, grafting raised both leaf concentrations of micronutrients and crop yield [6]. Micronutrients are involved in many metabolic and cellular functions essential to plant growth, such as energy metabolism, synthesis of primary and secondary metabolites, hormonal balance, and signal transduction [38].

Despite the higher leaf B concentrations in SP sour passion fruit, in both irrigation water salinities (Figure 4A), it was not enough according to the nutritional requirements of the plant (39 to 47 mg kg^−1^), as reported by [35]. López-Gómez et al. [22] described similar results for grafted loquat (*Eriobotrya japonica* Lindl.) under salt stress and fertilized with B. These authors reported that leaf B concentrations increase in grafted plants, reducing lipid peroxidation by salt stress and improving cell membrane protection.

Plastic mulching minimizes soil water losses through evaporation [39]. Under such a situation, sour passion fruit plants had lower leaf Cl^−^ concentrations (Figure 4B). Therefore, higher moisture in the soil irrigated with moderately saline water reduced soluble salts, such as chloride, in the topsoil layer, wherein absorbing roots are significantly concentrated [32,40]. The application of plastic mulching is important in conditions where the water has high concentrations of Cl^−^ ion. In this case, the absorption of this ion is accompanied by a decrease in the concentration of N-NO_3_^-^ in the aerial parts of the plants [17].

Based on leaf concentrations of Na (Figure 4C) and other nutrients (Figure 1, Figure 2 and Figure 3) in both SP and GP plants, as rootstock, *P. cincinnata* acts as a filter of ions mobilized to tillers. Generally, species native to saline environments have saline stress tolerance genes that can be transmitted to commercial species to obtain more tolerant hybrids [41]. Ferreira et al. [42] observed that the genes involved in sodium transport (SOS1 and SOS3) were upregulated in sour passion fruit under a water salinity of 12 dS m^−1^. Another factor that also contributes to selectivity in salt absorption is the membrane transporters that regulate ionic homeostasis in cells, especially Na^+^/H^+^ and K^+^/H^+^, transporters of sucrose and amino acids [43].

Lima et al. [41] attributed the reduction of up to 50% of the foliar concentration of Na^+^ in *Passiflora mucronata* Lam compared to *P. edulis*, both irrigated with saline water (150 mM NaCl), to the possible presence of these genes in the wild species. Fanny irrigated tomato (*Lycopersicon esculentum* Mill) cv Pwith 60 mM NaCl water. The use of rootstock AR-9704 reduced foliar sodium concentration by 29.16% compared to non-grafted plants [5]. The use of grafting on citrus Cleopatra Mandarin (*Citrus reticulata* Blanco) on Alemow (*Citrus macrophylla*) irrigated with saline water reduced the presence of Na in the aerial parts by 63% in comparison with non-grafted plants [21]. Its ability to accumulate Na and excrete salts by roots, as already verified in other Passiflora species [11,42], acts as a retention mechanism and prevents damage to plant shoots [23,24], reducing Na concentrations in the leaf tissue of the scion.

For plants irrigated with moderately saline water and grown in mulched soil (Figure 5A), fruit production overcame the maximum of 10.76 kg plant^−1^, observed by [8], for sour passion fruit irrigated with saline water and fertilized with bovine biofertilizer. Soil plastic mulching promoted a higher increase in fruit production of plants irrigated with moderately saline water (+95.7%) than irrigated with low salinity water (+33.7%). This finding highlights the benefits of plastic mulching by maintaining irrigated water volume, suitable edaphic microclimate, and reducing toxic salts within the soil layer below the root zone [31,44].

The root system absorbs water and nutrients from the soil and is the organ that is most affected under limiting conditions, such as low water availability or high levels of toxic ions [27,45]. Thus, plastic mulching in soil promoted favorable conditions for the absorption of water and nutrients and increased the production of sour passion fruit.

Fruit production in SP plants was always higher than in GP plants (Figure 5B). These results are similar to those reported by [46], who evaluated the productive capacity of sour passion fruit propagated by cutting and grafting on sweet passion fruit (*Passiflora alata*) and passion fruit Giberti (*Passiflora gibertii*). The authors verified that grafted plants were less vigorous during vegetative growth, forming lighter fruits [14]. According to [47], higher fruit productions in non-grafted plants are due to an increased average mass of harvested fruits than in grafted plants, as observed in our study (SP = 240.7 g and GP = 204.6 g—results not presented). Despite the greater accumulation of nutrients and reduction of foliar Na^+^ and Cl^−^ in the grafted plants, this was not reflected in fruit production and is due to the loss of vigor of the grafted plants observed in the field over time. This demonstrates that propagation by grafting, a technique recently used in passion fruit, still requires more investigations related to the grafting material and the most appropriate technique.

## 4. Materials and Methods

### 4.1. Characterization of the Experimental Area

**Figure 6:**
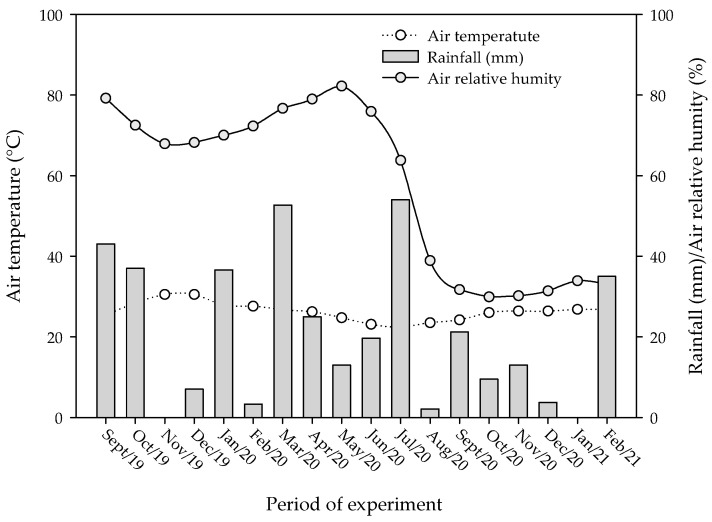
Meteorological data—temperature, relative humidity of the air, and rainfall collected at the experimental site during the study period.

The experiment was carried out in an experimental area located at ‘Macaquinhos Farm’, in the municipality of Remígio (7°00′1.95″ S, 35°47′55″ W and 562-m above sea level), Paraíba State, Brazil, between September 2019 and February 2021. According to Köppen’s classification, the local climate is classified as As’ type, which means that it is tropical hot and humid and has a dry season in winter [48]. The average air temperature, relative air humidity, and rainfall during the experimental period were 26.3 °C, 57.6%, and 375.8 mm, respectively (Figure 6).

The soil of the experimental area (0–0.40 m) was classified, according to the criteria of the [49], as arenic *Psamment*. Before the installation of the experiment, soil samples were collected in the area, mixed. Then, a composite sample was analyzed for chemical (fertility and salinity) and physical analyses according to [50], as presented in Table 3.

### 4.2. Experimental Design and Plant Material Used

**Figure 7:**
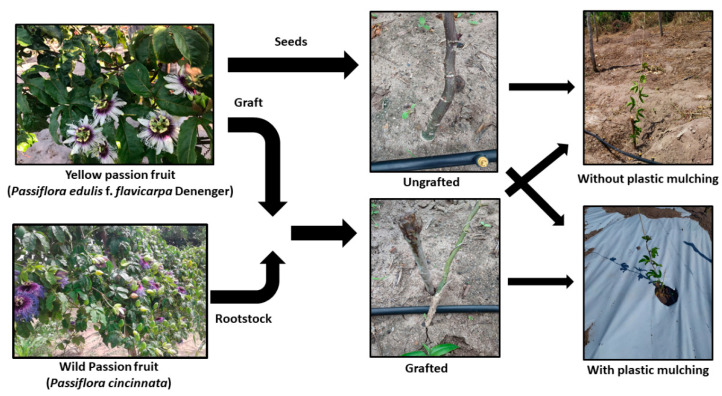
Experimental design of sour passion fruit propagated by seeds (SP) and grafted on *P. cincinnata* (GP) irrigated with low salinity (0.5 dS m^−1^) and moderately saline (4.5 dS m^−1^) irrigation water and grown in plastic-mulched and bare soil.

The experimental design was in randomized blocks and split plots in a 2 × (2 × 2) factorial scheme. The main plots were represented by low salinity (0.5 dS m^−1^) and moderately saline (4.5 dS m^−1^) irrigation water. The subplots were sour passion fruit propagated by seeds (SP) and grafted on wild passion fruit (GP) grown in plastic-mulched and bare soil (without mulch) conditions (Figure 7), with four replicates and three plants per plot.

Seed-propagated seedlings of the sour passion fruit accession ‘Guinezinho’ (SP) and seedlings grafted on wild passion fruit (*Passiflora cincinnata*) (GP) were evaluated in the experiment. The choice of passion fruits ‘Guinezinho’ and *Passiflora cincinnata* is due to the proven tolerance of plant materials to biotic stress and saline environments, respectively, compared to commercial varieties and wild species [9,51]. Seeds of non-grafted seedlings were collected in an orchard near the experimental area from fruits at the physiological maturation stage [52]. The scion variety was obtained from tertiary branches at the vegetative stage of plants in an orchard near the experimental area (Figure 1). Rootstock variety was obtained from seeds collected from fruits of plants grown in the municipality of Cerro Corá, in Rio Grande do Norte (6°2′45″ S, 36°20′45″ W), Brazil (Figure 7). The grafting technique employed was the full cleft, on the rootstocks, 90 days after sowing (DAS).

### 4.3. Experiment Installation and Performance

Holes were dug and measured 0.40 × 0.40 × 0.40 m (64 dm^3^), separating soil from the 0–0.20 and 0.20–0.40 m depth layers. To the 0–0.20 m soil layer, 20 L well-decomposed cattle manure (Table 4) and 50 g FTE-BR12 fertilizer (S = 3.9%, B = 1.8%, Cu = 0.85%, Mn = 2.0%, and Zn = 9.0%) [53] were added for fertilization, as well as 120 g dolomitic limestone (CaO = 47%, MgO = 3.4%, and RPTN = 82%) to raise soil base saturation to 70%. It was then immediately returned to the hole.

Passion fruit vines were trained on the espalier system, using smooth wire #12 fixed on 2.30-m high stakes buried 0.30 m into the ground and spaced 3 m apart. At the end of the line, the stake diameter was 0.20 m. This was to withstand the tension of the training system and plants. The planting spacing was 3 m between plants and 2 m between rows, totaling 1667 ha^−1^ [54]. Seedlings were transplanted when they reached from 0.25 to 0.30 m in height and had four fully expanded leaf pairs.

Low salinity water (0.5 dS m^−1^) was collected from a surface dam near the experimental area, and moderately saline water (4.5 dS m^−1^) was obtained by dissolving non-iodinated NaCl (94% purity) into the low salinity water (Table 5). Electrical conductivity was measured using a portable Instrutherm model CD-850 conductivity meter. Over the first 30 days after transplanting (DAT), plants were irrigated with low salinity water (0.5 dS m^−1^) to allow root system establishment.

Afterwards, plants were irrigated according to each treatment to replace evapotranspiration losses. The crop evapotranspiration (**ETc**) was estimated as the product of potential evapotranspiration (**ET_0_**) and crop coefficient (**kc**), according to methods described by [56,57]:

(1) $$ETc=ET_{0}\times kc$$

Crop coefficients adopted were 0.69 for the vegetative stage, 0.82 for flowering, and 1.09 for fruiting [58]. A Class-A tank was installed near the experimental area, and its evaporation (**ETa**) was used to determine ET_0_ by multiplying **ET_a_** by a correction coefficient (0.75), as suggested by [59]:

(2) $$ETa=ET_{a}\times 0.75$$

For irrigation, a drip system was used. The system was installed before the seedling transplanting, and the soil was covered with plastic mulch. Four pressure auto-compensating drippers were used for each plant (two facing east and two facing west at 0.20 and 0.40 m apart from the plant stem, respectively). The system was set to a flow rate and service pressure of 4 L h^−1^ and 0.2 MPa, respectively.

The soil was covered with a 320-µ-thick white plastic film to protect the soil (mulching) under the three plants in treated plots. The plastic film dimensions were 2.0 m wide and 12 m long, and it was fixed at a distance of 2 m between rows, covering a surface of 24.0 m^2^. At the points where the seedlings were transplanted, 0.40-m diameter holes were dug. Then, the unprotected area was covered with a plastic sheet to prevent evaporation.

Nitrogen (N), phosphorus (P), and potassium (K) topdressings were performed through fertigation using a Venturi injector [55]. N and K were supplied every 15 days at a ratio of 1:1 as urea (45% N) and potassium sulfate (50% K_2_O and 45% S). Phosphorus was supplied monthly as mono-ammonium phosphate—MAP (50% P_2_O_5_ and 10% N). Micronutrients (boron [B], copper [Cu], iron [Fe], manganese [Mn], molybdenum [Mo], and zinc [Zn]) were applied via foliar fertilization following recommendations of [60].

### 4.4. Traits Analyzed

#### 4.4.1. Plant Nutritional Status

At the full flowering stage (120 DAT), in the treatments of each block (four blocks), eight intact and healthy leaves were sampled from the middle part of sour passion fruit plants: four to the east and four to the west from the third or fourth leaf pairs. According to the recommendation of [61], for the sour passion fruit, leaf sampling is carried out at the time of full bloom, as this is the phase with the highest nutritional demand for the crop, and its purpose is to guide possible corrections in fertilization. The samples were analyzed for nutritional status in terms of macro and micronutrients, as well as sodium per dry matter weight [62]. The determination of the nutritional status of the plants was carried out as follows: nitrogen (N) by the Kjeldahl method (wet digestion); phosphorus (P) by molybdenum blue spectrometry; potassium (K) and sodium (Na) by atomic emission spectroscopy; calcium (Ca), magnesium (Mg), sulfur (S), copper (Cu), and iron (Fe) using an atomic absorption spectrophotometer at wavelengths of 422.7, 285.2, 400.0, 3274.7, and 508.0 nm, respectively; boron (B) by UV–vis spectrophotometry at a wavelength of 460.0 nm; manganese (Mn) zinc (Zn) by flame-acetylene atomic absorption spectrometry; and, chloride (Cl^−^) by the volumetric method of Mohr [63].

#### 4.4.2. Fruit Production per Plant

Fruits were harvested daily as their peels turned predominantly yellow, which occurred 60 days after anthesis [52]. The harvested fruits were counted and weighed on an electronic scale to calculate production per plant (kg per plant).

### 4.5. Statistical Analysis

Data were subjected to analysis of variance (ANOVA) by the F-test at a 0.05 probability level, after performing a test for normality and data homogeneity using the Shapiro–Wilk test. The means referring to the sources of variation and the interactions were compared by the Tukey test (*p* > 0.05). Data were analyzed using the statistical software SISVAR 5.6 [64].

## 5. Conclusions

Our results point out that, as rootstock, *Passiflora cincinnata* can alleviate harmful effects of water salinity on sour passion fruit plants increasing absorption of nutrients (K, Ca, Mg, S, Fe, Mn, and Zn) and restricting sodium absorption or transport to the scion variety, but without positive effects on fruit production. Mulching with plastic film, by reducing the presence of toxic salts close to the root zone, promoted greater absorption of elements such as N and Mg and reduced Na and Cl, contributing to greater production of sour passion fruit. Sour passion fruit propagated by seeds and grafted accumulate foliar macronutrients in the following order: N > Ca > K > Mg > S > P; and micronutrients and sodium: Na > Fe > Zn > Mn > B > Cl > Cu (seeds) and Na > Fe > Zn > B > Mn > Cl > Cu (grafted). The use of plastic mulch film in sour passion fruit irrigated with moderately saline water reduced leaf Na^+^ and Cl^−^ concentrations and increased production per plant compared to bare soil. The results in fruit production suggest that plastic mulch attenuates the effects of salts and increases the production capacity of sour passion fruit plants, with an emphasis on seed-propagated plants. Even though *Passiflora cincinnata* rootstock increased absorption of nutrients and decreased sodium concentrations in leaf tissue, it was not reflected in high fruit yields due to loss of production vigor. For future studies, we suggest that studies related to the biochemical and molecular activity of sour passion fruit grafted on *Passiflora cincinnata* be investigated to elucidate possible tolerance mechanisms present in wild species and how they are transferred to commercial cultivars.

## Figures and Tables

**Table 1:** Variance analysis summary and mean concentrations of nitrogen (N), phosphorus (P), potassium (K), calcium (Ca), magnesium (Mg), and sulfur (S) in sour passion fruit leaves as a function of water salinity (WS), plant propagation (Pg), and plastic mulching (PM).

Source of Variation	N	P	K	Ca	Mg	S
	g kg^−1^					
Water salinity (WS)						
Low salinity (0.5 dS m^−1^)	38.6 a	2.1 a	9.2 a	14.0 a	3.8 a	2.7 a
Moderately saline (4.5 dS m^−1^)	37.7 a	2.1 a	10.9 a	14.6 a	3.5 b	2.9 a
Propagation (Pg)						
Seed (SP)	37.8 a	2.2 a	8.7 b	12.6 b	3.1 b	2.7 a
Grafting (GP)	38.8 a	2.0 b	11.4 a	16.1 a	4.2 a	2.9 a
Plastic mulching (PM)						
Without	37.3 b	2.0 b	10.7 a	15.5 a	3.8 b	3.0 a
With	39.3 a	2.2 a	9.4 b	13.2 b	3.4 a	2.6 b
Analysis of variance mean squares						
WS × Pg	7.04 ^ns^	0.18 ^ns^	5.04 **	73.5 **	2.0 *	1.04 **
WS × PM	1.04 ^ns^	0.03 ^ns^	5.04 **	6.0 ^ns^	0.04 ^ns^	0.37 ^ns^
Pg × PM	12.04 ^ns^	0.12 **	22.04 **	88.2 **	2.0 *	0.04 ^ns^
WS × Pg × PM	5.04 ^ns^	0.33 ^ns^	0.37 ^ns^	8.2 *	1.04 ^ns^	0.04 ^ns^
Mean	38.3	2.1	10.0	14.3	3.6	2.8
CV1 (%)	5.6	6.75	31.7	9.9	0.1	12.7
CV2 (%)	4.2	4.6	6.4	7.9	13.8	11.9

CV = Coefficient of variation; ns, * and ** = not significant, significant at 0.05 and 0.01 probability level by the F-test, respectively; (a and b) means with equal letters do not differ from each other by the ‘Tukey’ test for water salinity, propagation and plastic mulching, respectively.

**Table 2:** Variance analysis summary and mean concentrations of copper (Cu), iron (Fe), manganese (Mn), zinc (Zn), boron (B), chlorine (Cl), and sodium (Na) in leaves of sour passion fruit plants as a function of water salinity (WS), plant propagation method (Pg), and plastic mulching (PM).

SV	Cu	Fe	Mn	Zn	B	Cl	Na
	mg kg^−1^						
Water salinity (WS)							
Low salinity (0.5 dS m^−1^)	6.5 a	184.6 a	32.8 a	49.8 a	34.8 b	13.8 b	1151 b
Moderately saline (4.5 dS m^−1^)	3.8 b	202.8 a	33.8 a	47.7 a	30.7 a	21.3 a	6327 a
Propagation (Pg)							
Seed (SP)	5.8 a	192.9 a	33.2 a	51.3 a	36.3 a	19.4 a	6671 a
Grafting (GP)	4.5 b	194.5 a	33.4 a	46.1 b	29.1 b	15.8 b	807 b
Plastic mulching (PM)							
Without	5.7 a	231.1 a	33.9 a	56.9 a	33.6 a	22.4 a	4750 a
With	4.6 b	156.2 b	32.7 a	40.6 b	31.8 a	12.8 b	2728 b
Analysis of variance mean squares							
WS × Pg	32.7 **	27,405 **	651 **	3504 **	0.37 ns	2.7 ns	1302 × 106 **
WS × PM	8.2 **	3675 ns	273 *	80 *	84.4 ns	216 **	167 × 106 **
							
Pg × PM	20.2 **	6633 *	376 *	522 **	247 **	20.2 ns	130 × 106 **
WS × Pg × PM	8.2 **	22,632 **	360 *	1872 **	22 ns	8.2 ns	156 × 106 **
Mean	5.2	193.7	33.3	48.8	32.7	17.6	3739.4
CV1 (%)	3.95	23.73	21.77	4.83	14.07	2.01	1.97
CV2 (%)	13.69	15.66	20.01	6.20	14.03	15.55	2.74

CV = Coefficient of variation; ns, *, and ** = non-significant, significant at 0.05, and significant at 0.01 probability level by the F-test, respectively; (a and b) means with equal letters do not differ from each other by the ‘Tukey’ test for water salinity, propagation and plastic mulching, respectively.

**Table 3:** Chemical (fertility and salinity) and physical properties of the soil (0–0.40 m depth) of the experimental area before the installation of experiment.

Soil Fertility		Soil Salinity		Soil Physical Properties	
pH	6.00	pHsp (H_2_O)	6.16	Sand (g kg^−1^)	831.5
P (mg dm^−3^)	16.63	EC (dS m^−1^)	0.22	Silt (g kg^−1^)	100.0
K^+^ (cmol_c_ dm^−3^)	0.08	SO_4_^2−^ (mmol_c_ L^−1^)	3.91	Clay (g kg^−1^)	68.5
Ca^2+^ (cmol_c_ dm^−3^)	1.09	Ca^2+^ (mmol_c_ L^−1^)	5.12	DW (g (kg^−1^)	0.00
Mg^2+^ (cmol_c_ dm^−3^)	1.12	Mg^2+^ (mmol_c_ L^−1^)	15.25	FD (kg dm^−3^)	1000
Na^+^ (cmol_c_ dm^−3^)	0.05	K^+^ (mmol_c_ L^−1^)	0.89	SD (g cm^−3^)	1.53
SB (cmol_c_ dm^−3^)	2.34	Na^+^ (mmol_c_ L^−1^)	5.70	PD (g cm^−3^)	2.61
H^+^ + Al^3+^ (cmol_c_ dm^−3^)	1.24	CO_3_^2−^ (mmol_c_ L^−1^)		TP (m^3^ m^−3^)	0.42
Al^3+^ (cmol_c_ dm^−3^)	0	Cl^−^ (mmol_c_ L^−1^)	15.00	H0.01 MPa (g kg^−1^)	65
CEC (cmol_c_ dm^−3^)	3.58	SAR (mmol L^−1^)^0,5^	0.28	H0.03 MPa (g kg^−1^)	49
V (%)	65.36	ESP (%)	1.39	H1.50 MPa (g kg^−1^)	28
OM (g kg^−1^)	13.58	Classification	Non saline non sodic	Textural class	Loamy sand

SB—Sum of bases (K^+^ + Ca^2+^ + Mg^2+^+ N a^+^); CEC—Cation exchange capacity (K^+^ + Ca^2+^ + Mg^2+^ + Na^+^+ [H^+^+Al^3+^]); V—Base saturation ([SB/CEC] × 100); OM—Organic matter; EC—Electric conductivity in 1:2 soil water suspension; SAR—Sodium adsorption ratio; ESP—Exchangeable sodium percentage; AD—Dispersed clay; FD—Flocculation degree; SD—Soil density; PD—Particle density; TP—Total porosity; U0.01MPa—Soil moisture at field capacity; U0.03MPa—Soil moisture at 80% field capacity; U1.5MPa—Soil moisture at permanent wilting point.

**Table 4:** Chemical characterization of the bovine manure used in the experiment.

Macronutrients		Micronutrients	
Organic carbon (g kg^−1^)	159.1	Boron (mg kg^−1^)	58.0
Nitrogen (g kg^−1^)	8.3	Copper (mg kg^−1^)	941.0
Carbon: nitrogen ratio	19.17	Iron (mg kg^−1^)	250.0
Phosphorus (g kg^−1^)	19.2	Manganese (mg kg^−1^)	8.0
Potassium (g kg^−1^)	10.4	Zinc (mg kg^−1^)	21.3
Calcium (g kg^−1^)	8.2	Sodium (mg kg^−1^)	79.0
Magnesium (g kg^−1^)	5.0	Hydrogen potential (H_2_O)	8.81
Sulfur (g kg^−1^)	1.8		

**Table 5:** Chemical characteristics of surface dam water used for irrigation with low salinity water (0.5 dS m^−1^) and to prepare moderately saline water (4.5 dS m^−1^).

EC	pH	K^+^	Ca^2+^	Mg^2+^	Na^+^	Cl^−^	CO_3_^2−^	SO_4_^2−^	SAR	Classification
dS m^−1^		mmol_c_ L^−1^							(mmol L^−1^)^1/2^	
0.5	6.10	0.28	0.65	0.27	1.88	1.87	0.00	0.51	2.77	C1S1

EC = electrical conductivity at 25 °C; C1S1 = Low risk of salinization and sodification of the soil, according to [55].
